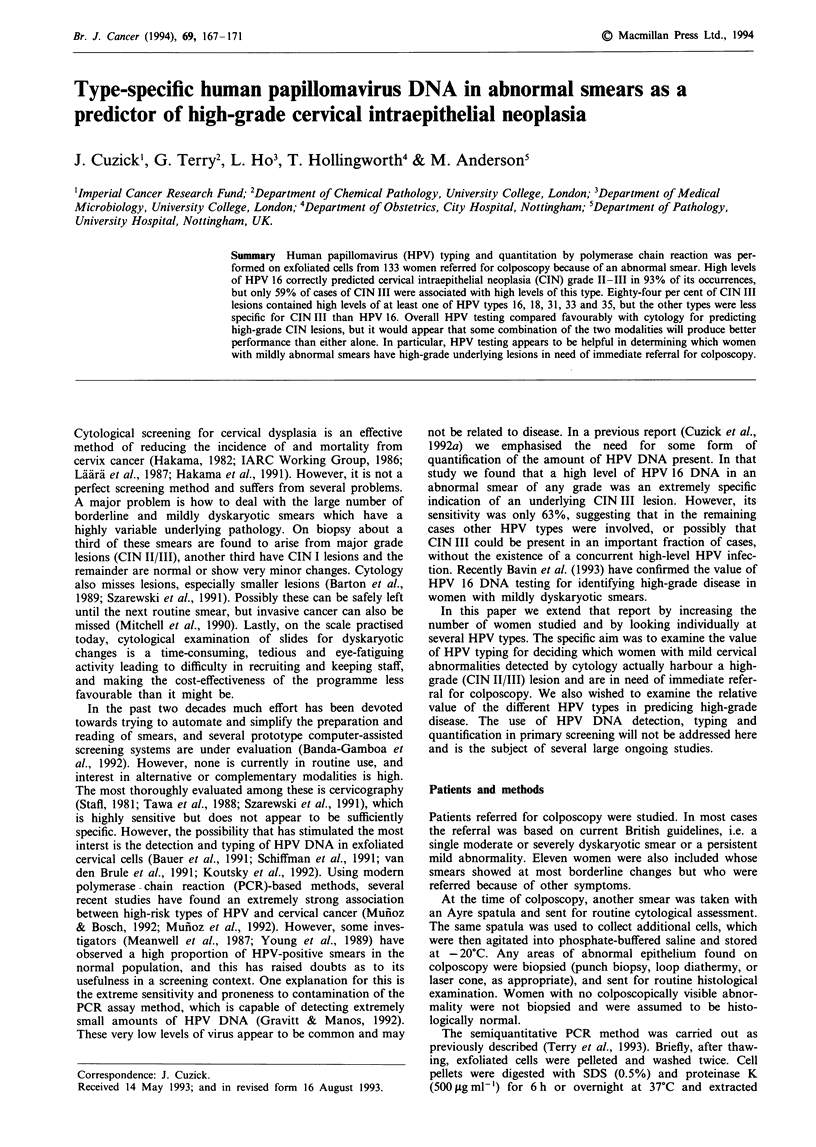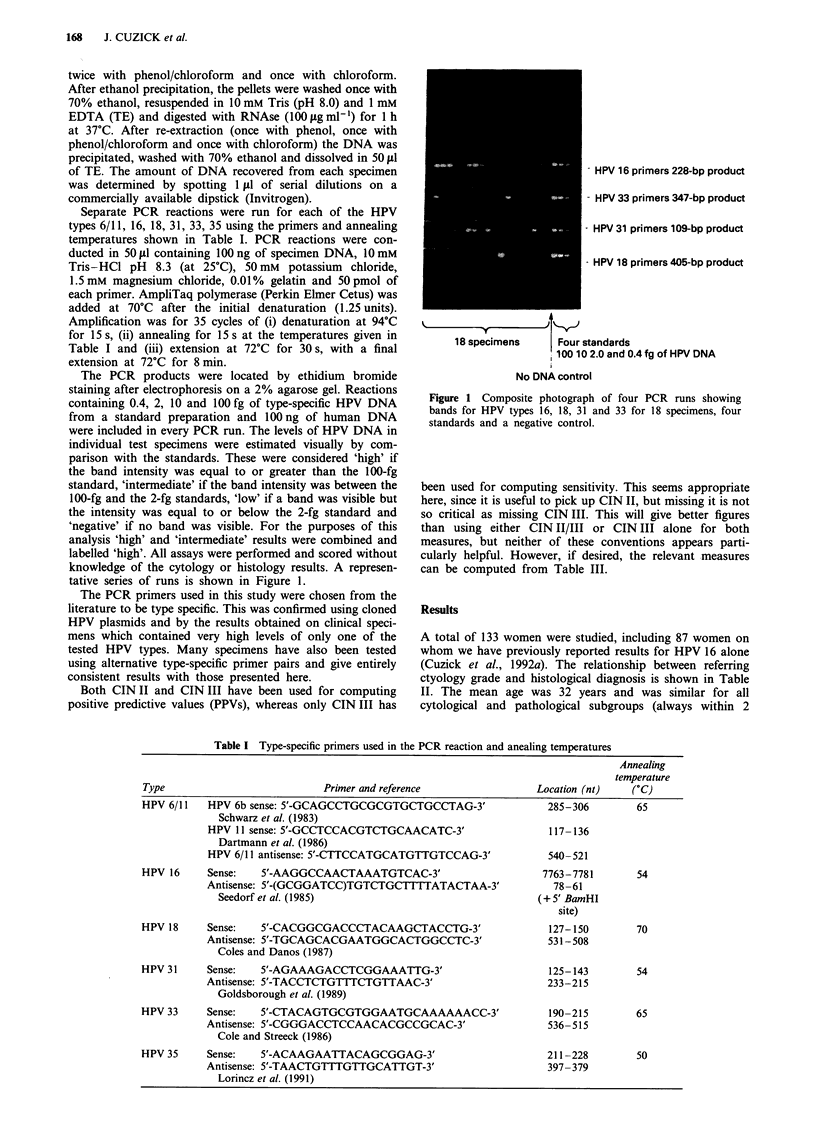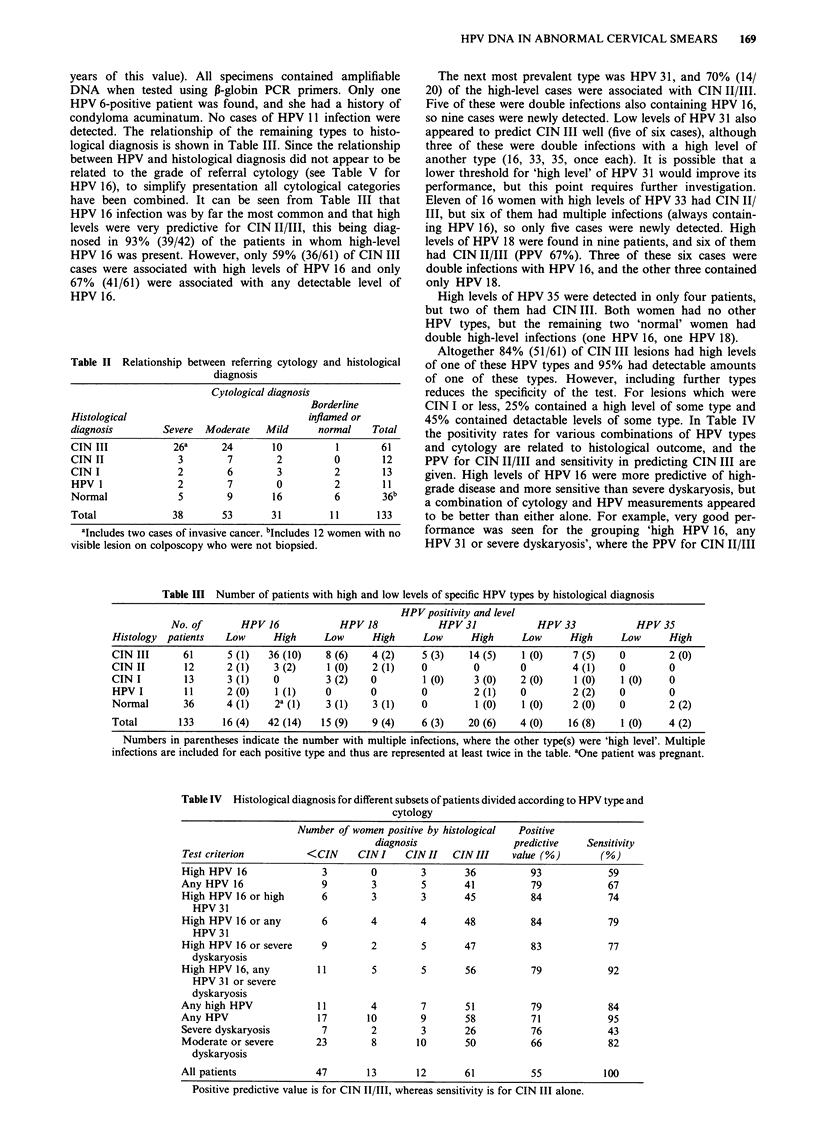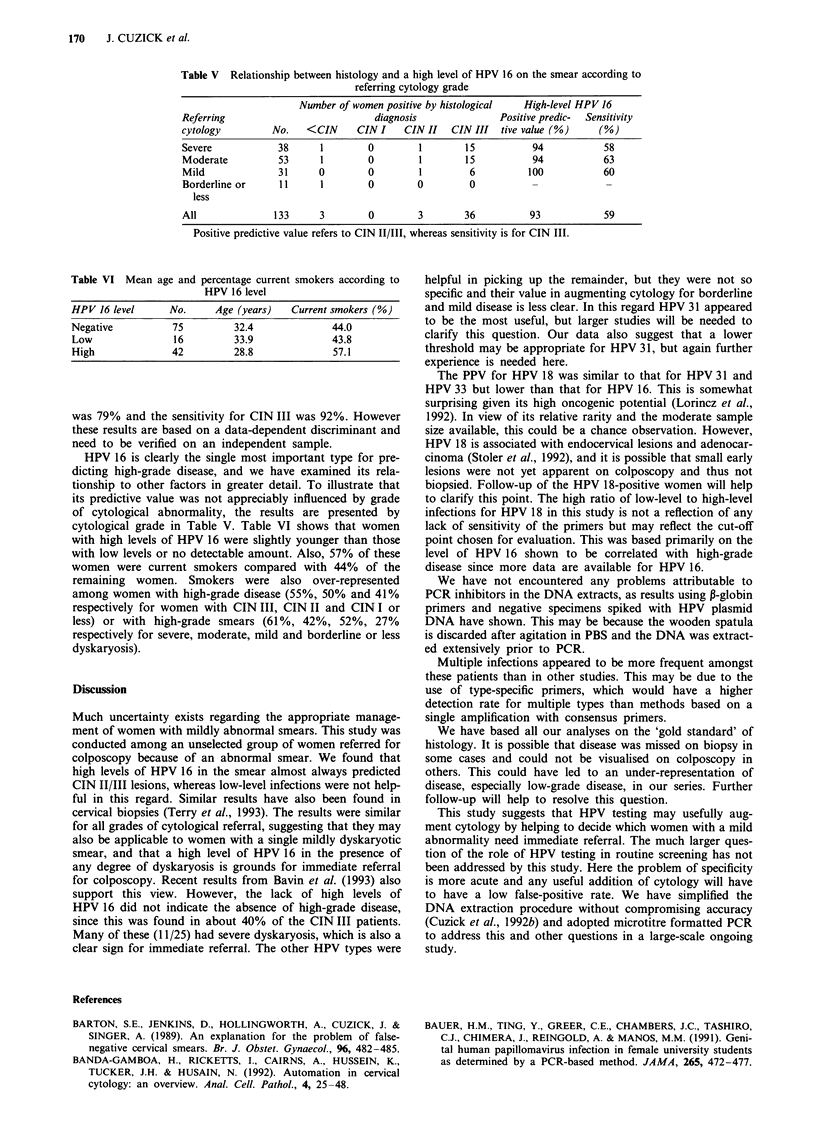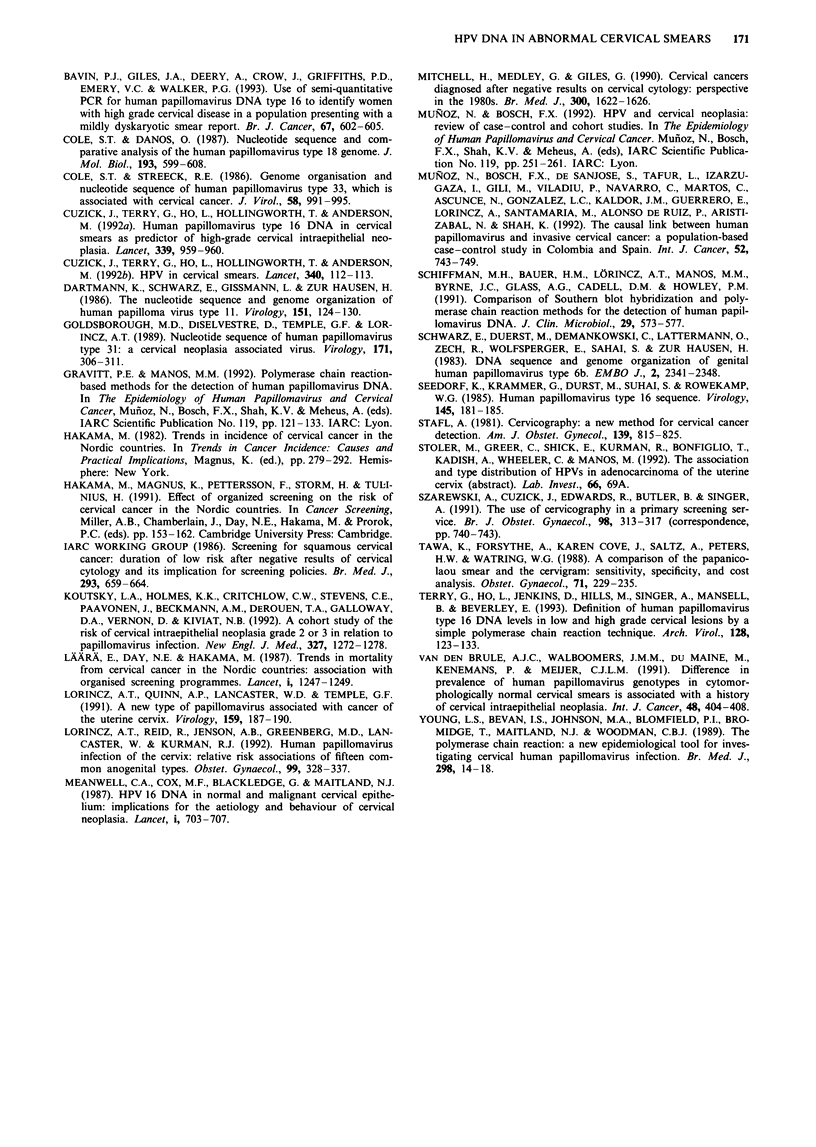# Type-specific human papillomavirus DNA in abnormal smears as a predictor of high-grade cervical intraepithelial neoplasia.

**DOI:** 10.1038/bjc.1994.28

**Published:** 1994-01

**Authors:** J. Cuzick, G. Terry, L. Ho, T. Hollingworth, M. Anderson

**Affiliations:** Imperial Cancer Research Fund, London, UK.

## Abstract

**Images:**


Br. J. Cancer (1994), 69, 167-171 © Macmillan Press Ltd., 1994

### Type-specific human papillomavirus DNA in abnormal smears as a predictor of high-grade cervical intraepithelial neoplasia

J. Cuzick[1], G. Terry[2], L. Ho[3], T. Hollingworth[4] & M. Anderson[5]

[1]*Imperial Cancer Research Fund;* [2]*Department of Chemical Pathology, University College, London;* [3]*Department of Medical Microbiology, University College, London;* [4]*Department of Obstetrics, City Hospital, Nottingham;* [5]*Department of Pathology, University Hospital, Nottingham, UK.*

**Summary** Human papillomavirus (HPV) typing and quantitation by polymerase chain reaction was performed on exfoliated cells from 133 women referred for colposcopy because of an abnormal smear. High levels of HPV 16 correctly predicted cervical intraepithelial neoplasia (CIN) grade II-III in 93% of its occurrences, but only 59% of cases of CIN III were associated with high levels of this type. Eighty-four per cent of CIN III lesions contained high levels of at least one of HPV types 16, 18, 31, 33 and 35, but the other types were less specific for CIN III than HPV 16. Overall HPV testing compared favourably with cytology for predicting high-grade CIN lesions, but it would appear that some combination of the two modalities will produce better performance than either alone. In particular, HPV testing appears to be helpful in determining which women with mildly abnormal smears have high-grade underlying lesions in need of immediate referral for colposcopy.

Cytological screening for cervical dysplasia is an effective method of reducing the incidence of and mortality from cervix cancer (Hakama, 1982; IARC Working Group, 1986; Läärä *et al.*, 1987; Hakama *et al.*, 1991). However, it is not a perfect screening method and suffers from several problems. A major problem is how to deal with the large number of borderline and mildly dyskaryotic smears which have a highly variable underlying pathology. On biopsy about a third of these smears are found to arise from major grade lesions (CIN II/III), another third have CIN I lesions and the remainder are normal or show very minor changes. Cytology also misses lesions, especially smaller lesions (Barton *et al.*, 1989; Szarewski *et al.*, 1991). Possibly these can be safely left until the next routine smear, but invasive cancer can also be missed (Mitchell *et al.*, 1990). Lastly, on the scale practised today, cytological examination of slides for dyskaryotic changes is a time-consuming, tedious and eye-fatiguing activity leading to difficulty in recruiting and keeping staff, and making the cost-effectiveness of the programme less favourable than it might be.

In the past two decades much effort has been devoted towards trying to automate and simplify the preparation and reading of smears, and several prototype computer-assisted screening systems are under evaluation (Banda-Gamboa *et al.*, 1992). However, none is currently in routine use, and interest in alternative or complementary modalities is high. The most thoroughly evaluated among these is cervicography (Stafl, 1981; Tawa *et al.*, 1988; Szarewski *et al.*, 1991), which is highly sensitive but does not appear to be sufficiently specific. However, the possibility that has stimulated the most interst is the detection and typing of HPV DNA in exfoliated cervical cells (Bauer *et al.*, 1991; Schiffman *et al.*, 1991; van den Brule *et al.*, 1991; Koutsky *et al.*, 1992). Using modern polymerase chain reaction (PCR)-based methods, several recent studies have found an extremely strong association between high-risk types of HPV and cervical cancer (Muñoz & Bosch, 1992; Muñoz *et al.*, 1992). However, some investigators (Meanwell *et al.*, 1987; Young *et al.*, 1989) have observed a high proportion of HPV-positive smears in the normal population, and this has raised doubts as to its usefulness in a screening context. One explanation for this is the extreme sensitivity and proneness to contamination of the PCR assay method, which is capable of detecting extremely small amounts of HPV DNA (Gravitt & Manos, 1992). These very low levels of virus appear to be common and may not be related to disease. In a previous report (Cuzick *et al.*, 1992*a*) we emphasised the need for some form of quantification of the amount of HPV DNA present. In that study we found that a high level of HPV 16 DNA in an abnormal smear of any grade was an extremely specific indication of an underlying CIN III lesion. However, its sensitivity was only 63%, suggesting that in the remaining cases other HPV types were involved, or possibly that CIN III could be present in an important fraction of cases, without the existence of a concurrent high-level HPV infection. Recently Bavin *et al.* (1993) have confirmed the value of HPV 16 DNA testing for identifying high-grade disease in women with mildly dyskaryotic smears.

In this paper we extend that report by increasing the number of women studied and by looking individually at several HPV types. The specific aim was to examine the value of HPV typing for deciding which women with mild cervical abnormalities detected by cytology actually harbour a high-grade (CIN II/III) lesion and are in need of immediate referral for colposcopy. We also wished to examine the relative value of the different HPV types in predicing high-grade disease. The use of HPV DNA detection, typing and quantification in primary screening will not be addressed here and is the subject of several large ongoing studies.

### Patients and methods

Patients referred for colposcopy were studied. In most cases the referral was based on current British guidelines, i.e. a single moderate or severely dyskaryotic smear or a persistent mild abnormality. Eleven women were also included whose smears showed at most borderline changes but who were referred because of other symptoms.

At the time of colposcopy, another smear was taken with an Ayre spatula and sent for routine cytological assessment. The same spatula was used to collect additional cells, which were then agitated into phosphate-buffered saline and stored at −20°C. Any areas of abnormal epithelium found on colposcopy were biopsied (punch biopsy, loop diathermy, or laser cone, as appropriate), and sent for routine histological examination. Women with no colposcopically visible abnormality were not biopsied and were assumed to be histologically normal.

The semiquantitative PCR method was carried out as previously described (Terry *et al.*, 1993). Briefly, after thawing, exfoliated cells were pelleted and washed twice. Cell pellets were digested with SDS (0.5%) and proteinase K (500 $\mu g\,ml^{-1}$) for 6 h or overnight at 37°C and extracted

Correspondence: J. Cuzick.
Received 14 May 1993; and in revised form 16 August 1993.

twice with phenol/chloroform and once with chloroform. After ethanol precipitation, the pellets were washed once with 70% ethanol, resuspended in 10 mM Tris (pH 8.0) and 1 mM EDTA (TE) and digested with RNAse (100 $\mu$g ml$^{-1}$) for 1 h at 37°C. After re-extraction (once with phenol, once with phenol/chloroform and once with chloroform) the DNA was precipitated, washed with 70% ethanol and dissolved in 50 $\mu$l of TE. The amount of DNA recovered from each specimen was determined by spotting 1 $\mu$l of serial dilutions on a commercially available dipstick (Invitrogen).

Separate PCR reactions were run for each of the HPV types 6/11, 16, 18, 31, 33, 35 using the primers and annealing temperatures shown in Table I. PCR reactions were conducted in 50 $\mu$l containing 100 ng of specimen DNA, 10 mM Tris-HCl pH 8.3 (at 25°C), 50 mM potassium chloride, 1.5 mM magnesium chloride, 0.01% gelatin and 50 pmol of each primer. AmpliTaq polymerase (Perkin Elmer Cetus) was added at 70°C after the initial denaturation (1.25 units). Amplification was for 35 cycles of (i) denaturation at 94°C for 15 s, (ii) annealing for 15 s at the temperatures given in Table I and (iii) extension at 72°C for 30 s, with a final extension at 72°C for 8 min.

The PCR products were located by ethidium bromide staining after electrophoresis on a 2% agarose gel. Reactions containing 0.4, 2, 10 and 100 fg of type-specific HPV DNA from a standard preparation and 100 ng of human DNA were included in every PCR run. The levels of HPV DNA in individual test specimens were estimated visually by comparison with the standards. These were considered 'high' if the band intensity was equal to or greater than the 100-fg standard, 'intermediate' if the band intensity was between the 100-fg and the 2-fg standards, 'low' if a band was visible but the intensity was equal to or below the 2-fg standard and 'negative' if no band was visible. For the purposes of this analysis 'high' and 'intermediate' results were combined and labelled 'high'. All assays were performed and scored without knowledge of the cytology or histology results. A representative series of runs is shown in Figure 1.

The PCR primers used in this study were chosen from the literature to be type specific. This was confirmed using cloned HPV plasmids and by the results obtained on clinical specimens which contained very high levels of only one of the tested HPV types. Many specimens have also been tested using alternative type-specific primer pairs and give entirely consistent results with those presented here.

Both CIN II and CIN III have been used for computing positive predictive values (PPVs), whereas only CIN III has been used for computing sensitivity. This seems appropriate here, since it is useful to pick up CIN II, but missing it is not so critical as missing CIN III. This will give better figures than using either CIN II/III or CIN III alone for both measures, but neither of these conventions appears particularly helpful. However, if desired, the relevant measures can be computed from Table III.

**Figure 1** Composite photograph of four PCR runs showing bands for HPV types 16, 18, 31 and 33 for 18 specimens, four standards and a negative control.

### Results

A total of 133 women were studied, including 87 women on whom we have previously reported results for HPV 16 alone (Cuzick *et al.*, 1992*a*). The relationship between referring ctyology grade and histological diagnosis is shown in Table II. The mean age was 32 years and was similar for all cytological and pathological subgroups (always within 2

**Table I** Type-specific primers used in the PCR reaction and anealing temperatures

| *Type* | *Primer and reference* | *Location (nt)* | *Annealing temperature (°C)* |
|---|---|---|---|
| HPV 6/11 | HPV 6b sense: 5′-GCAGCCTGCGCGTGCTGCCTAG-3′ | 285–306 | 65 |
| | Schwarz *et al.* (1983) | | |
| | HPV 11 sense: 5′-GCCTCCACGTCTGCAACATC-3′ | 117–136 | |
| | Dartmann *et al.* (1986) | | |
| | HPV 6/11 antisense: 5′-CTTCCATGCATGTTGTCCAG-3′ | 540–521 | |
| HPV 16 | Sense: 5′-AAGGCCAACTAAATGTCAC-3′ | 7763–7781 | 54 |
| | Antisense: 5′-(GCGGATCC)TGTCTGCTTTTATACTAA-3′ | 78–61 | |
| | Seedorf *et al.* (1985) | (+5′ *Bam*HI site) | |
| HPV 18 | Sense: 5′-CACGGCGACCCTACAAGCTACCTG-3′ | 127–150 | 70 |
| | Antisense: 5′-TGCAGCACGAATGGCACTGGCCTC-3′ | 531–508 | |
| | Coles and Danos (1987) | | |
| HPV 31 | Sense: 5′-AGAAAGACCTCGGAAATTG-3′ | 125–143 | 54 |
| | Antisense: 5′-TACCTCTGTTTCTGTTAAC-3′ | 233–215 | |
| | Goldsborough *et al.* (1989) | | |
| HPV 33 | Sense: 5′-CTACAGTGCGTGGAATGCAAAAAACC-3′ | 190–215 | 65 |
| | Antisense: 5′-CGGGACCTCCAACACGCCGCAC-3′ | 536–515 | |
| | Cole and Streeck (1986) | | |
| HPV 35 | Sense: 5′-ACAAGAATTACAGCGGAG-3′ | 211–228 | 50 |
| | Antisense: 5′-TAACTGTTTGTTGCATTGT-3′ | 397–379 | |
| | Lorincz *et al.* (1991) | | |

years of this value). All specimens contained amplifiable DNA when tested using β-globin PCR primers. Only one HPV 6-positive patient was found, and she had a history of condyloma acuminatum. No cases of HPV 11 infection were detected. The relationship of the remaining types to histological diagnosis is shown in Table III. Since the relationship between HPV and histological diagnosis did not appear to be related to the grade of referral cytology (see Table V for HPV 16), to simplify presentation all cytological categories have been combined. It can be seen from Table III that HPV 16 infection was by far the most common and that high levels were very predictive for CIN II/III, this being diagnosed in 93% (39/42) of the patients in whom high-level HPV 16 was present. However, only 59% (36/61) of CIN III cases were associated with high levels of HPV 16 and only 67% (41/61) were associated with any detectable level of HPV 16.

**Table II** Relationship between referring cytology and histological diagnosis

| Histological diagnosis | *Cytological diagnosis* Severe | Moderate | Mild | Borderline inflamed or normal | Total |
|---|---|---|---|---|---|
| CIN III | 26[a] | 24 | 10 | 1 | 61 |
| CIN II | 3 | 7 | 2 | 0 | 12 |
| CIN I | 2 | 6 | 3 | 2 | 13 |
| HPV 1 | 2 | 7 | 0 | 2 | 11 |
| Normal | 5 | 9 | 16 | 6 | 36[b] |
| Total | 38 | 53 | 31 | 11 | 133 |

[a]Includes two cases of invasive cancer. [b]Includes 12 women with no visible lesion on colposcopy who were not biopsied.

The next most prevalent type was HPV 31, and 70% (14/20) of the high-level cases were associated with CIN II/III. Five of these were double infections also containing HPV 16, so nine cases were newly detected. Low levels of HPV 31 also appeared to predict CIN III well (five of six cases), although three of these were double infections with a high level of another type (16, 33, 35, once each). It is possible that a lower threshold for 'high level' of HPV 31 would improve its performance, but this point requires further investigation. Eleven of 16 women with high levels of HPV 33 had CIN II/III, but six of them had multiple infections (always containing HPV 16), so only five cases were newly detected. High levels of HPV 18 were found in nine patients, and six of them had CIN II/III (PPV 67%). Three of these six cases were double infections with HPV 16, and the other three contained only HPV 18.

High levels of HPV 35 were detected in only four patients, but two of them had CIN III. Both women had no other HPV types, but the remaining two 'normal' women had double high-level infections (one HPV 16, one HPV 18).

Altogether 84% (51/61) of CIN III lesions had high levels of one of these HPV types and 95% had detectable amounts of one of these types. However, including further types reduces the specificity of the test. For lesions which were CIN I or less, 25% contained a high level of some type and 45% contained detectable levels of some type. In Table IV the positivity rates for various combinations of HPV types and cytology are related to histological outcome, and the PPV for CIN II/III and sensitivity in predicting CIN III are given. High levels of HPV 16 were more predictive of high-grade disease and more sensitive than severe dyskaryosis, but a combination of cytology and HPV measurements appeared to be better than either alone. For example, very good performance was seen for the grouping 'high HPV 16, any HPV 31 or severe dyskaryosis', where the PPV for CIN II/III

**Table III** Number of patients with high and low levels of specific HPV types by histological diagnosis

| Histology | No. of patients | *HPV positivity and level* HPV 16 Low | HPV 16 High | HPV 18 Low | HPV 18 High | HPV 31 Low | HPV 31 High | HPV 33 Low | HPV 33 High | HPV 35 Low | HPV 35 High |
|---|---|---|---|---|---|---|---|---|---|---|---|
| CIN III | 61 | 5 (1) | 36 (10) | 8 (6) | 4 (2) | 5 (3) | 14 (5) | 1 (0) | 7 (5) | 0 | 2 (0) |
| CIN II | 12 | 2 (1) | 3 (2) | 1 (0) | 2 (1) | 0 | 0 | 0 | 4 (1) | 0 | 0 |
| CIN I | 13 | 3 (1) | 0 | 3 (2) | 0 | 1 (0) | 3 (0) | 2 (0) | 1 (0) | 1 (0) | 0 |
| HPV I | 11 | 2 (0) | 1 (1) | 0 | 0 | 0 | 2 (1) | 0 | 2 (2) | 0 | 0 |
| Normal | 36 | 4 (1) | 2[a] (1) | 3 (1) | 3 (1) | 0 | 1 (0) | 1 (0) | 2 (0) | 0 | 2 (2) |
| Total | 133 | 16 (4) | 42 (14) | 15 (9) | 9 (4) | 6 (3) | 20 (6) | 4 (0) | 16 (8) | 1 (0) | 4 (2) |

Numbers in parentheses indicate the number with multiple infections, where the other type(s) were 'high level'. Multiple infections are included for each positive type and thus are represented at least twice in the table. [a]One patient was pregnant.

**Table IV** Histological diagnosis for different subsets of patients divided according to HPV type and cytology

| Test criterion | *Number of women positive by histological diagnosis* <CIN | CIN I | CIN II | CIN III | Positive predictive value (%) | Sensitivity (%) |
|---|---|---|---|---|---|---|
| High HPV 16 | 3 | 0 | 3 | 36 | 93 | 59 |
| Any HPV 16 | 9 | 3 | 5 | 41 | 79 | 67 |
| High HPV 16 or high HPV 31 | 6 | 3 | 3 | 45 | 84 | 74 |
| High HPV 16 or any HPV 31 | 6 | 4 | 4 | 48 | 84 | 79 |
| High HPV 16 or severe dyskaryosis | 9 | 2 | 5 | 47 | 83 | 77 |
| High HPV 16, any HPV 31 or severe dyskaryosis | 11 | 5 | 5 | 56 | 79 | 92 |
| Any high HPV | 11 | 4 | 7 | 51 | 79 | 84 |
| Any HPV | 17 | 10 | 9 | 58 | 71 | 95 |
| Severe dyskaryosis | 7 | 2 | 3 | 26 | 76 | 43 |
| Moderate or severe dyskaryosis | 23 | 8 | 10 | 50 | 66 | 82 |
| All patients | 47 | 13 | 12 | 61 | 55 | 100 |

Positive predictive value is for CIN II/III, whereas sensitivity is for CIN III alone.

**Table V** Relationship between histology and a high level of HPV 16 on the smear according to referring cytology grade

| Referring cytology | No. | Number of women positive by histological diagnosis <CIN | CIN I | CIN II | CIN III | High-level HPV 16 Positive predictive value (%) | Sensitivity (%) |
|---|---|---|---|---|---|---|---|
| Severe | 38 | 1 | 0 | 1 | 15 | 94 | 58 |
| Moderate | 53 | 1 | 0 | 1 | 15 | 94 | 63 |
| Mild | 31 | 0 | 0 | 1 | 6 | 100 | 60 |
| Borderline or less | 11 | 1 | 0 | 0 | 0 | – | – |
| All | 133 | 3 | 0 | 3 | 36 | 93 | 59 |

Positive predictive value refers to CIN II/III, whereas sensitivity is for CIN III.

**Table VI** Mean age and percentage current smokers according to HPV 16 level

| HPV 16 level | No. | Age (years) | Current smokers (%) |
|---|---|---|---|
| Negative | 75 | 32.4 | 44.0 |
| Low | 16 | 33.9 | 43.8 |
| High | 42 | 28.8 | 57.1 |

was 79% and the sensitivity for CIN III was 92%. However these results are based on a data-dependent discriminant and need to be verified on an independent sample.

HPV 16 is clearly the single most important type for predicting high-grade disease, and we have examined its relationship to other factors in greater detail. To illustrate that its predictive value was not appreciably influenced by grade of cytological abnormality, the results are presented by cytological grade in Table V. Table VI shows that women with high levels of HPV 16 were slightly younger than those with low levels or no detectable amount. Also, 57% of these women were current smokers compared with 44% of the remaining women. Smokers were also over-represented among women with high-grade disease (55%, 50% and 41% respectively for women with CIN III, CIN II and CIN I or less) or with high-grade smears (61%, 42%, 52%, 27% respectively for severe, moderate, mild and borderline or less dyskaryosis).

### Discussion

Much uncertainty exists regarding the appropriate management of women with mildly abnormal smears. This study was conducted among an unselected group of women referred for colposcopy because of an abnormal smear. We found that high levels of HPV 16 in the smear almost always predicted CIN II/III lesions, whereas low-level infections were not helpful in this regard. Similar results have also been found in cervical biopsies (Terry *et al.*, 1993). The results were similar for all grades of cytological referral, suggesting that they may also be applicable to women with a single mildly dyskaryotic smear, and that a high level of HPV 16 in the presence of any degree of dyskaryosis is grounds for immediate referral for colposcopy. Recent results from Bavin *et al.* (1993) also support this view. However, the lack of high levels of HPV 16 did not indicate the absence of high-grade disease, since this was found in about 40% of the CIN III patients. Many of these (11/25) had severe dyskaryosis, which is also a clear sign for immediate referral. The other HPV types were helpful in picking up the remainder, but they were not so specific and their value in augmenting cytology for borderline and mild disease is less clear. In this regard HPV 31 appeared to be the most useful, but larger studies will be needed to clarify this question. Our data also suggest that a lower threshold may be appropriate for HPV 31, but again further experience is needed here.

The PPV for HPV 18 was similar to that for HPV 31 and HPV 33 but lower than that for HPV 16. This is somewhat surprising given its high oncogenic potential (Lorincz *et al.*, 1992). In view of its relative rarity and the moderate sample size available, this could be a chance observation. However, HPV 18 is associated with endocervical lesions and adenocarcinoma (Stoler *et al.*, 1992), and it is possible that small early lesions were not yet apparent on colposcopy and thus not biopsied. Follow-up of the HPV 18-positive women will help to clarify this point. The high ratio of low-level to high-level infections for HPV 18 in this study is not a reflection of any lack of sensitivity of the primers but may reflect the cut-off point chosen for evaluation. This was based primarily on the level of HPV 16 shown to be correlated with high-grade disease since more data are available for HPV 16.

We have not encountered any problems attributable to PCR inhibitors in the DNA extracts, as results using β-globin primers and negative specimens spiked with HPV plasmid DNA have shown. This may be because the wooden spatula is discarded after agitation in PBS and the DNA was extracted extensively prior to PCR.

Multiple infections appeared to be more frequent amongst these patients than in other studies. This may be due to the use of type-specific primers, which would have a higher detection rate for multiple types than methods based on a single amplification with consensus primers.

We have based all our analyses on the 'gold standard' of histology. It is possible that disease was missed on biopsy in some cases and could not be visualised on colposcopy in others. This could have led to an under-representation of disease, especially low-grade disease, in our series. Further follow-up will help to resolve this question.

This study suggests that HPV testing may usefully augment cytology by helping to decide which women with a mild abnormality need immediate referral. The much larger question of the role of HPV testing in routine screening has not been addressed by this study. Here the problem of specificity is more acute and any useful addition of cytology will have to have a low false-positive rate. We have simplified the DNA extraction procedure without compromising accuracy (Cuzick *et al.*, 1992*b*) and adopted microtitre formatted PCR to address this and other questions in a large-scale ongoing study.

### References

BARTON, S.E., JENKINS, D., HOLLINGWORTH, A., CUZICK, J. & SINGER, A. (1989). An explanation for the problem of false-negative cervical smears. *Br. J. Obstet. Gynaecol.*, **96**, 482–485.

BANDA-GAMBOA, H., RICKETTS, I., CAIRNS, A., HUSSEIN, K., TUCKER, J.H. & HUSAIN, N. (1992). Automation in cervical cytology: an overview. *Anal. Cell. Pathol.*, **4**, 25–48.

BAUER, H.M., TING, Y., GREER, C.E., CHAMBERS, J.C., TASHIRO, C.J., CHIMERA, J., REINGOLD, A. & MANOS, M.M. (1991). Genital human papillomavirus infection in female university students as determined by a PCR-based method. *JAMA*, **265**, 472–477.

BAVIN, P.J., GILES, J.A., DEERY, A., CROW, J., GRIFFITHS, P.D., EMERY, V.C. & WALKER, P.G. (1993). Use of semi-quantitative PCR for human papillomavirus DNA type 16 to identify women with high grade cervical disease in a population presenting with a mildly dyskaryotic smear report. *Br. J. Cancer*, **67**, 602–605.

COLE, S.T. & DANOS, O. (1987). Nucleotide sequence and comparative analysis of the human papillomavirus type 18 genome. *J. Mol. Biol.*, **193**, 599–608.

COLE, S.T. & STREECK, R.E. (1986). Genome organisation and nucleotide sequence of human papillomavirus type 33, which is associated with cervical cancer. *J. Virol.*, **58**, 991–995.

CUZICK, J., TERRY, G., HO, L., HOLLINGWORTH, T. & ANDERSON, M. (1992*a*). Human papillomavirus type 16 DNA in cervical smears as predictor of high-grade cervical intraepithelial neoplasia. *Lancet*, **339**, 959–960.

CUZICK, J., TERRY, G., HO, L., HOLLINGWORTH, T. & ANDERSON, M. (1992*b*). HPV in cervical smears. *Lancet*, **340**, 112–113.

DARTMANN, K., SCHWARZ, E., GISSMANN, L. & ZUR HAUSEN, H. (1986). The nucleotide sequence and genome organization of human papilloma virus type 11. *Virology*, **151**, 124–130.

GOLDSBOROUGH, M.D., DISELVESTRE, D., TEMPLE, G.F. & LORINCZ, A.T. (1989). Nucleotide sequence of human papillomavirus type 31: a cervical neoplasia associated virus. *Virology*, **171**, 306–311.

GRAVITT, P.E. & MANOS, M.M. (1992). Polymerase chain reaction-based methods for the detection of human papillomavirus DNA. In *The Epidemiology of Human Papillomavirus and Cervical Cancer*, Muñoz, N., Bosch, F.X., Shah, K.V. & Meheus, A. (eds). IARC Scientific Publication No. 119, pp. 121–133. IARC: Lyon.

HAKAMA, M. (1982). Trends in incidence of cervical cancer in the Nordic countries. In *Trends in Cancer Incidence: Causes and Practical Implications*, Magnus, K. (ed.), pp. 279–292. Hemisphere: New York.

HAKAMA, M., MAGNUS, K., PETTERSSON, F., STORM, H. & TULINIUS, H. (1991). Effect of organized screening on the risk of cervical cancer in the Nordic countries. In *Cancer Screening*, Miller, A.B., Chamberlain, J., Day, N.E., Hakama, M. & Prorok, P.C. (eds). pp. 153–162. Cambridge University Press: Cambridge.

IARC WORKING GROUP (1986). Screening for squamous cervical cancer: duration of low risk after negative results of cervical cytology and its implication for screening policies. *Br. Med. J.*, **293**, 659–664.

KOUTSKY, L.A., HOLMES, K.K., CRITCHLOW, C.W., STEVENS, C.E., PAAVONEN, J., BECKMANN, A.M., DEROUEN, T.A., GALLOWAY, D.A., VERNON, D. & KIVIAT, N.B. (1992). A cohort study of the risk of cervical intraepithelial neoplasia grade 2 or 3 in relation to papillomavirus infection. *New Engl. J. Med.*, **327**, 1272–1278.

LÄÄRÄ, E., DAY, N.E. & HAKAMA, M. (1987). Trends in mortality from cervical cancer in the Nordic countries: association with organised screening programmes. *Lancet*, **i**, 1247–1249.

LORINCZ, A.T., QUINN, A.P., LANCASTER, W.D. & TEMPLE, G.F. (1991). A new type of papillomavirus associated with cancer of the uterine cervix. *Virology*, **159**, 187–190.

LORINCZ, A.T., REID, R., JENSON, A.B., GREENBERG, M.D., LANCASTER, W. & KURMAN, R.J. (1992). Human papillomavirus infection of the cervix: relative risk associations of fifteen common anogenital types. *Obstet. Gynaecol.*, **99**, 328–337.

MEANWELL, C.A., COX, M.F., BLACKLEDGE, G. & MAITLAND, N.J. (1987). HPV 16 DNA in normal and malignant cervical epithelium: implications for the aetiology and behaviour of cervical neoplasia. *Lancet*, **i**, 703–707.

MITCHELL, H., MEDLEY, G. & GILES, G. (1990). Cervical cancers diagnosed after negative results on cervical cytology: perspective in the 1980s. *Br. Med. J.*, **300**, 1622–1626.

MUÑOZ, N. & BOSCH, F.X. (1992). HPV and cervical neoplasia: review of case–control and cohort studies. In *The Epidemiology of Human Papillomavirus and Cervical Cancer*. Muñoz, N., Bosch, F.X., Shah, K.V. & Meheus, A. (eds), IARC Scientific Publication No. 119, pp. 251–261. IARC: Lyon.

MUÑOZ, N., BOSCH, F.X., DE SANJOSE, S., TAFUR, L., IZARZUGAZA, I., GILI, M., VILADIU, P., NAVARRO, C., MARTOS, C., ASCUNCE, N., GONZALEZ, L.C., KALDOR, J.M., GUERRERO, E., LORINCZ, A., SANTAMARIA, M., ALONSO DE RUIZ, P., ARISTIZABAL, N. & SHAH, K. (1992). The causal link between human papillomavirus and invasive cervical cancer: a population-based case–control study in Colombia and Spain. *Int. J. Cancer*, **52**, 743–749.

SCHIFFMAN, M.H., BAUER, H.M., LÖRINCZ, A.T., MANOS, M.M., BYRNE, J.C., GLASS, A.G., CADELL, D.M. & HOWLEY, P.M. (1991). Comparison of Southern blot hybridization and polymerase chain reaction methods for the detection of human papillomavirus DNA. *J. Clin. Microbiol.*, **29**, 573–577.

SCHWARZ, E., DUERST, M., DEMANKOWSKI, C., LATTERMANN, O., ZECH, R., WOLFSPERGER, E., SAHAI, S. & ZUR HAUSEN, H. (1983). DNA sequence and genome organization of genital human papillomavirus type 6b. *EMBO J.*, **2**, 2341–2348.

SEEDORF, K., KRAMMER, G., DURST, M., SUHAI, S. & ROWEKAMP, W.G. (1985). Human papillomavirus type 16 sequence. *Virology*, **145**, 181–185.

STAFL, A. (1981). Cervicography: a new method for cervical cancer detection. *Am. J. Obstet. Gynecol.*, **139**, 815–825.

STOLER, M., GREER, C., SHICK, E., KURMAN, R., BONFIGLIO, T., KADISH, A., WHEELER, C. & MANOS, M. (1992). The association and type distribution of HPVs in adenocarcinoma of the uterine cervix (abstract). *Lab. Invest.*, **66**, 69A.

SZAREWSKI, A., CUZICK, J., EDWARDS, R., BUTLER, B. & SINGER, A. (1991). The use of cervicography in a primary screening service. *Br. J. Obstet. Gynaecol.*, **98**, 313–317 (correspondence, pp. 740–743).

TAWA, K., FORSYTHE, A., KAREN COVE, J., SALTZ, A., PETERS, H.W. & WATRING, W.G. (1988). A comparison of the papanicolaou smear and the cervigram: sensitivity, specificity, and cost analysis. *Obstet. Gynaecol.*, **71**, 229–235.

TERRY, G., HO, L., JENKINS, D., HILLS, M., SINGER, A., MANSELL, B. & BEVERLEY, E. (1993). Definition of human papillomavirus type 16 DNA levels in low and high grade cervical lesions by a simple polymerase chain reaction technique. *Arch. Virol.*, **128**, 123–133.

VAN DEN BRULE, A.J.C., WALBOOMERS, J.M.M., DU MAINE, M., KENEMANS, P. & MEIJER, C.J.L.M. (1991). Difference in prevalence of human papillomavirus genotypes in cytomorphologically normal cervical smears is associated with a history of cervical intraepithelial neoplasia. *Int. J. Cancer*, **48**, 404–408.

YOUNG, L.S., BEVAN, I.S., JOHNSON, M.A., BLOMFIELD, P.I., BROMIDGE, T., MAITLAND, N.J. & WOODMAN, C.B.J. (1989). The polymerase chain reaction: a new epidemiological tool for investigating cervical human papillomavirus infection. *Br. Med. J.*, **298**, 14–18.